# Fabrication of flexible temperature sensors to explore indeterministic data analysis for robots as an application of Internet of Things

**DOI:** 10.1039/d2ra03015b

**Published:** 2022-06-09

**Authors:** Usama Afzal, Fatima Afzal, Kanza Maryam, Muhammad Aslam

**Affiliations:** Department of Physics, University of Education, Township Campus Lahore 54000 Pakistan mohammadusamafzal7@gmail.com; School of Chemistry, University of the Punjab Lahore 54000 Pakistan f.afzal.edu@gmail.com k.maryam.edu@gmail.com; Departments of Statistics, Faculty of Science, King Abdulaziz University Jeddah 21551 Saudi Arabia aslam_ravian@hotmail.com

## Abstract

The use of flexible electronic devices in different applications of Internet of Things, especially in robot technology, has gained importance to measure different physical factors such as temperature. Moreover, there is a need for a flexible and more informative approach to analyse the data. In this study, we report two flexible temperature sensors based on reduced graphene and multi-walled carbon nanotubes with high sensitivity and quick response and recovery times. The electrical properties of the sensors were studied using an LCR meter associated with a controlled chamber at 1 kHz. We used both classical and neutrosophic methods for analyzing the measured data of temperature sensors and found the more effective method by comparing their methods of analysis.

## Abstract 2

The use of flexible electronic devices in different applications of Internet of Things, especially in robot technology, has gained importance to measure different physical factors such as temperature.

## Graphical abstract

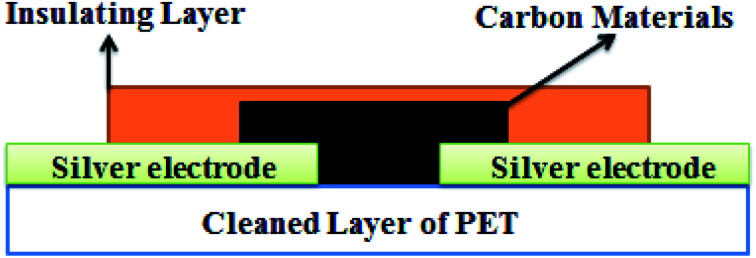

## 1. Introduction

Developments in information technology (IT) and Internet of Things (IoT) have been increasing since past few years. This is also a reason for increase in the fabrication of sensors,^1^ which are utilized to monitor different factors such as motion, humidity and temperature. Temperature is an important factor, which plays an important role in all the fields of life including monitoring the human body,^2^ electronic skin^3^ and robot body temperatures. Generally, all temperature sensors detect the change in the body temperature.^4^ On the basis of their functionality, the temperature sensors can be divided into four types, namely, thermocouples,^5^ thermistors,^6^ resistance temperature detectors^7^ and semiconductor-based integrated circuits.^8^ However, resistance temperature detectors are commonly used due to their remarkable properties such as stability, quick response and accuracy.^9^ For achieving high stability and accuracy, researchers have used different types of materials for the fabrication of these sensors. However, among the temperature-sensitive materials, carbon-based materials including graphenes,^10^ carbon nanotubes,^11^ black carbon^12^ and carbon fibers are commonly known due to their internal structure, which makes them highly sensitive to temperature. Flexible sensors are always desirable as they can accurately monitor the temperature of any artificial skin or surface.^13^ Accordingly, various structures have been used to fabricate flexible sensors. Currently, flexible sensors are fabricated using flexible polymer substrates such as polyimides,^14^ polyethylene terephthalates (PETs)^15^ and polydimethylsiloxanes (PDMSs).^16^ Such flexible sensors are widely used in robotic technology as robots are considered the future of IoT, and can be used in all fields of life.^17^ These sensors are attached to the skin or body of robots to sense the temperature,^18^ pressure^19^ and motion variance^20^ with respect to any incident. Consequently, such research topics have attracted the interest of researchers. In order to study the temperature variance in robots, the skin of the robot is considered a good frame of reference. Such data variance can be deterministic or indeterministic, *i.e.* single values with respect to some constants or interval values. If data are deterministic, classical formulas can analyze them well, and if data are indeterministic, the classical formulas fail to explain.

To overcome this problem, we have applied a novel statistical approach called neutrosophic that can accurately analyze both the deterministic and indeterministic data. Smarandache proposed this approach in 2013.^21^ It is a suitable, flexible and more informative statistical approach than all the other approaches including fuzzy and classical. Now researchers have turned to the neutrosophic approach to solve problems in medicine,^22^ applied science,^23^ astrophysics,^24^ material science,^25^*etc.* Further, Afzal *et al.* used a neutrosophic approach to analyze the capacitance and resistance of a humidity sensor.^26^ Moreover, Aslam also worked and proposed different techniques using neutrosophic statistics.^27,28^

In this work, we have fabricated a surface-type resistance temperature detector to measure the temperature of the robot skin. This sensor is based on the carbon-type material^29^ sensing film, which is deposited on a polyethylene terephthalate (PET) substrate between two silver electrodes. The structure, optical and surface properties of carbon materials were also studied using different techniques. The resistance variance with respect to change in temperature was measured and analyzed using an LCR meter. To the best of our knowledge, this is the first time that a controlled chamber with an LCR meter was used to measure the data of temperature sensors. Similarly, we used the classical and neutrosophic methods to analyze the measured data for resistance with respect to the change in the temperature of sensors (we have already used the classical and neutrosophic methods to analyze the resistance data of conductors^25^ and 3D graphenes^30^ with respect to the change in temperature).

## 2. Experimental

**Fig. 1:**
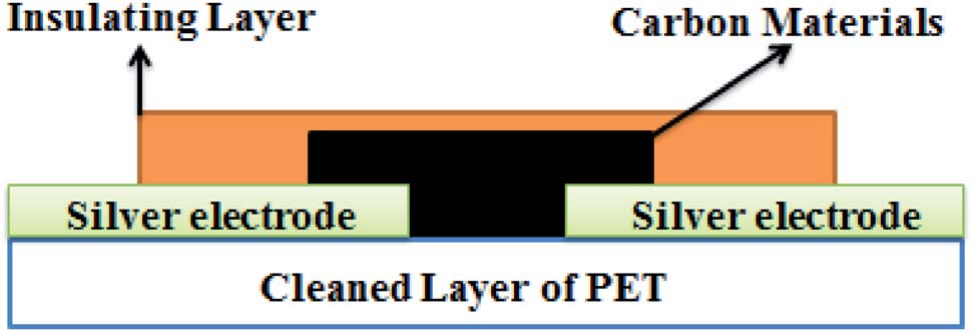
Schematic diagram of a flexible temperature sensor.

In this work, 99.9% pure carbon materials, namely, reduced graphene oxide (rGO) and multi-walled carbon nanotubes (MWCNTs) were used. First, two flexible PET substrates were cleaned with acetone and deionized water for fifteen minutes, separately. Then, two silver electrodes separated by 50 μm were deposited using a thermal evaporator at 10^−5^ mbar with a shadow mask on each. Then, the carbon materials (rGO and MWCNTs) were deposited separately between these electrodes using an air-spray coating technique. Next, an insulting thin layer was deposited on it by directly pressing the transparent tape on the sensing thin film area with 400 N force. In this way, two highly flexible and highly sensitive temperature sensors were fabricated: one based on the sensing film of rGO and another based on the sensing film of MWCNTs for the robot skin and device, as shown in Fig. 1.

**Fig. 2:**
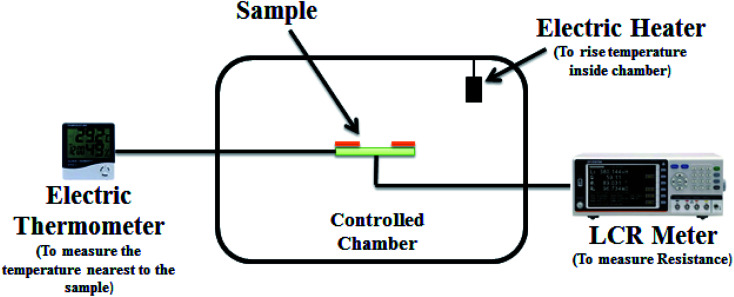
The setup for electrical characterization.

The structural properties of carbon materials were studied by X-ray diffraction, optical absorption through UV-Vis spectroscopy and surface morphology by scanning electron microscopy (SEM). Similarly, the electric properties of sensors, *i.e.* change in resistance with respect to the increase in temperature, were studied in the laboratory at room temperature of about 20 °C. The resistance of the sensors was measured using an LCR meter at 1 kHz associated with a control chamber, as shown in Fig. 2. All the readings of resistance variance with respect to the change in temperature were measured at intervals, which means at a specific point of temperature the minimum and maximum value change, *i.e.* [minimum value, maximum value]. Then we introduced the neutrosophic formula for the resistance variance due to the change in temperature. For analysis on resistance, both classical (conventional) and neutrosophic formulas were used.

## 3. Results and discussion

**Fig. 3:**
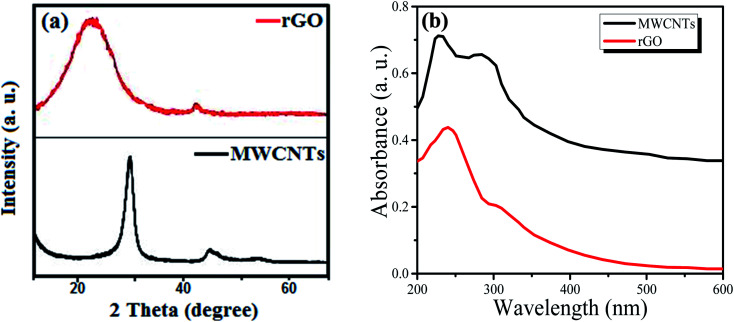
(a) XRD patterns of reduced graphene and multi-walled carbon nanotubes. (b) UV-Vis absorbance of reduced graphene and multi-walled carbon nanotubes.

**Fig. 4:**
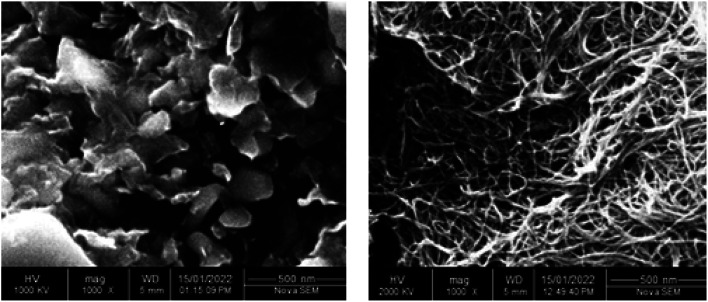
Left side: SEM image of rGO. Right side: SEM image of MWCNTs.

The structural properties of the carbon materials, *i.e.* rGO and MWCNTs, were observed *via* X-ray diffraction (XRD) technique with Cu Kα radiation (*λ* = 1.5406 Å) as the X-ray source, as shown in Fig. 3a. It is seen that for rGO there are two peaks, a broader peak and a small peak at 22.3° and 42.9° for the (002) and (102) planes, respectively, which are in accordance with the crystalline structure.^31^ Similarly, for MWCNTs, there are two peaks, a sharp peak and a small peak at 27.4° and 44.2° for the (002) and (100) planes, respectively. It is observed that the XRD pattern of MWCNTs is more similar to that of graphite due to its intrinsic nature.^32^ Similarly, the optical properties of the sensing films were studied by UV-Vis spectroscopy. Fig. 3b shows the UV-Vis absorption for rGO and MWCNTs. For rGO, the absorption spectrum shows a red-shift of the 235 nm peak to 290 nm because of the oxygen functional group removal and conjugate structure restoration.^33^ Similarly, for MWCNTs, the absorption spectrum shows a significance peak from 210 to 295 nm. It is observed that both the absorption spectra fall in the 200 to 300 nm region, which means that these carbon materials are good candidates for use in solar cells and light sensing applications. Similarly, the surface morphology of the carbon materials can be seen in Fig. 4. Both figures were observed at 1000× magnification using scanning electron microscopy (SEM).

The electrical properties of the sensor were studied, and are based on the resistance variance with respect to the change in temperature from 20 to 100 °C and measured after each 5 °C change in temperature using an LCR meter at 1 kHz (the maximum and minimum values of resistance change at the specific point of temperature, *i.e.* [minimum value; maximum value]). As already mentioned, all data were measured at intervals, because we also wanted to observe the resistance variance at a specific temperature value, as can be seen in Table 1.

**Table 1:** Measured resistance for both rGO and MWCNTs

Temperature (°C)	Resistance	
	rGO (kΩ)	MWCNTs (kΩ)
20	[4.499, 5.063]	[0.961, 0.971]
25	[4.296, 4.844]	[0.960, 0.970]
30	[4.079, 4.599]	[0.957, 0.967]
35	[3.885, 4.381]	[0.953, 0.963]
40	[3.716, 4.190]	[0.950, 0.960]
44	[3.499, 3.945]	[0.948, 0.958]
50	[3.282, 3.700]	[0.944, 0.954]
55	[3.136, 3.536]	[0.942, 0.952]
60	[3.040, 3.428]	[0.940, 0.950]
65	[2.894, 3.264]	[0.936, 0.946]
70	[2.798, 3.156]	[0.932, 0.942]
75	[2.750, 3.101]	[0.924, 0.938]
80	[2.653, 2.991]	[0.924, 0.934]
85	[2.557, 2.883]	[0.920, 0.930]
90	[2.388, 2.692]	[0.915, 0.925]
95	[2.266, 2.556]	[0.912, 0.922]
100	[2.194, 2.474]	[0.907, 0.917]

Table 1 presents the measured values of resistance of sensors based on rGO and MWNCTs with respect to the change in temperature from 20 to 100 °C. It can be observed that the resistance of the sensors decreased as the temperature of the chamber increased.

### 3.1. Temperature effect on sensor resistance

Several researchers have worked on the resistance change of sensors based on carbon materials (rGO and MWCNTs) with the change in temperature and found that resistance decreased with the increase in temperature^34,35^ due to their electric properties. Graphene is a good zero-bandgap semiconductor having no charge carriers at fermi level.^36^ Due to such semiconductor properties, the active charge carriers increase as the temperature increases. Thus, with the increase in charge carriers, the flow of current starts to increase, which increases the conductivity.^37^ As conductivity is inversely proportional to resistance, this leads to a decrease in the resistance. Thus, the resistance of the reduced graphene oxide used in temperature sensors decreases with the increase in the temperature of the chamber. This relation is expressed by the following equation:1
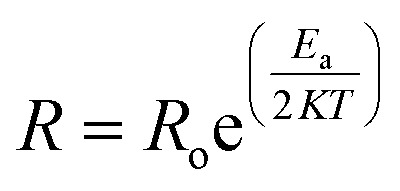
here,

*R* is the measured resistance of sensor.

*T* is the ambient temperature.

*R*_o_ is the resistance at the initial level.

*K* is the Boltzmann Constant.

*E*_a_ is the thermal activation.

A similar result was obtained with the resistance of the sensor based on MWCNTs. However, in the case of MWCNTs, there are multiple layers of carbon nanotubes, which is the reason for more current flow. Thus, the MWCNT-based temperature sensor has less resistance than the rGO-based temperature sensor.

### 3.2. Response and recovery time

**Fig. 5:**
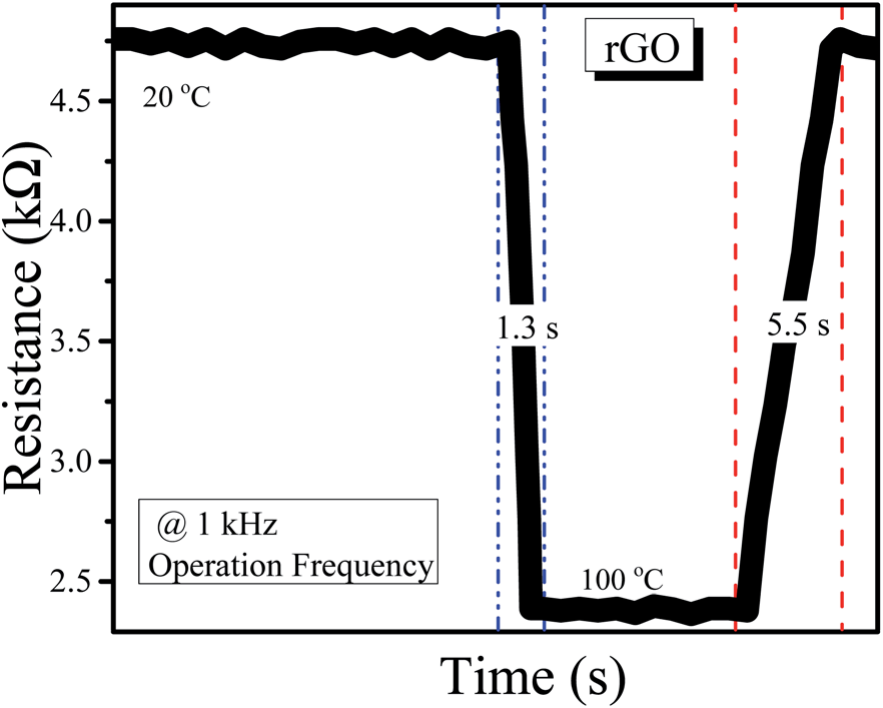
Response and recovery time for the rGO-based temperature sensor.

**Fig. 6:**
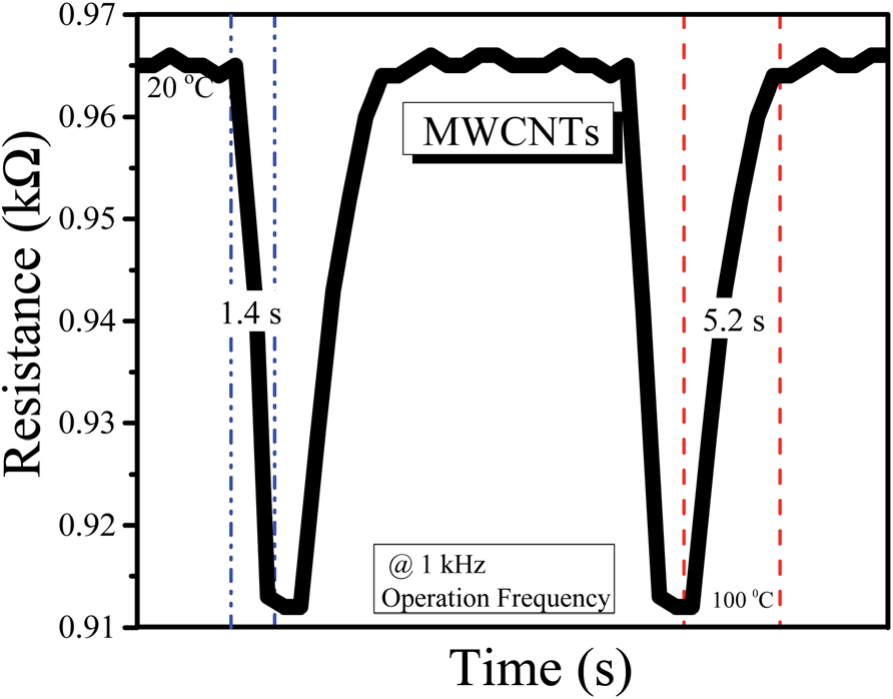
Response and recovery time for the MWCNT-based temperature sensor.

The response and recovery times of both the rGO-based and MWCNT-based temperature sensors are shown in Fig. 5 and 6. The response time is 1.3 s and the recovery time is 5.5 s for the sensor based on the rGO sensing film. Similarly, for MWCNTs, the response time is 1.4 s and the recovery time is 5.2 s.

### 3.3. Sensitivity of sensors

The sensitivity of sensors is a very important factor, which is measured using the following formula:2
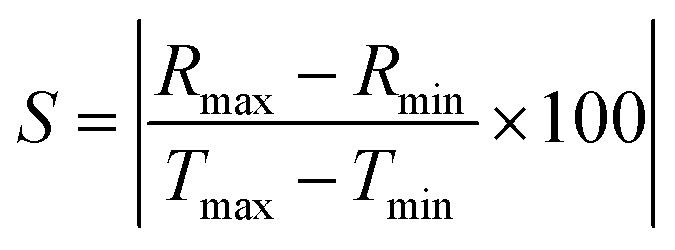
where ‘*R*_max_’ is the maximum resistance, ‘*R*_min_’ is the minimum resistance, ‘*T*_max_’ is the maximum temperature and ‘*T*_min_’ is the minimum temperature of sensors.

The sensitivity of rGO- and MWCNT-based sensors is 2.869% (kΩ °C^−1^) and 0.064% (kΩ °C^−1^), respectively. This sensitivity is based on the surface morphology of the sensing thin films. Reduced graphene has better surface properties than multi-walled carbon nanotubes, and hence, it has more sensitivity.

### 3.4. Use of sensors for steel body

**Fig. 7:**
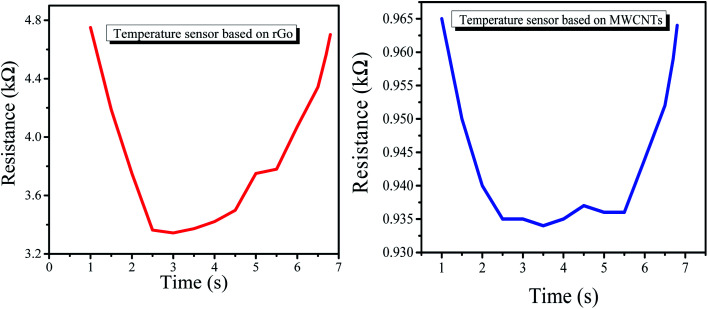
Resistance output of the temperature sensors.

We used the sensor to measure the temperature of a steel body. We used a steel glass and put the sensor on its wall surface. Initially, when the glass was empty, the sensor showed maximum resistance, and after pouring hot water (about 50 °C), the resistance of the sensor started to decrease, as the hot water increased the temperature of the steel glass wall. The whole experiment was performed at room temperature. The output plots are shown in Fig. 7.

### 3.5. Analysis of resistance variance

Next, the resistance of these sensors was analyzed using classical and neutrosophic formulas to obtain the resistance data. Basically, this is the extension of our previous work, namely, the analysis of conductors and 3D graphene resistance with respect to the change in temperature in material statistics (a study in which the data of material properties are analyzed by different methods of statistics) by applying classical and neutrosophic methods for analyzing the data of temperature sensors based on rGO and MWCNTs.

The classical analysis contains the average formula to calculate the values as shown below:^25^*R*(*T*)*i* = (*L*_*i*_ + *U*_*i*_)/2 *i* = 0, 1, 2, 3…*n*where ‘*L*’ and ‘*U*’ are the lower and upper values of each interval, respectively and *R*(*T*) is the resistance as a function of temperature, as the resistance of sensors depends on the temperature variance. However, in this way, we only obtained single values at a specific temperature for each step. For a large number of measurements, the computational algorithm to run on software is as follows:

Step 1: Start program

Step 2: Run loop *i* = 20 to *i* <= 100

Step 3: Execute formula: 
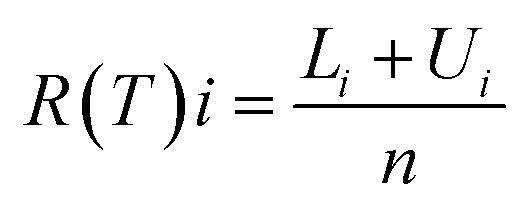


Calculate values and draw graph

Step 4: Increment of 5 and go to step 2

Step 5: End program

Similarly, for using the modern and novel neutrosophic approach, first, we have to develop the neutrosophic method (this task has been performed in our previous work,^25^ here, we only use the formula). When *R*(*T*)_*N*_ ∈ [*R*(*T*)_*L*_, *R*(*T*)_*U*_] is the measured interval of resistance with a lower value ‘*R*(*T*)_*L*_’ and an upper value ‘*R*(*T*)_*U*_’, the neutrosophic formula is given below:*R*(*T*)_*N*_ = *R*(*T*)_*L*_ + *R*(*T*)_*U*_*I*_*N*_; *I*_*N*_ ∈ [*I*_*L*_, *I*_*U*_]where *I*_*N*_ ∈ [*I*_*L*_, *I*_*U*_] is the indeterminacy interval. The above-mentioned neutrosophic formula is the extension of the classical formula as ‘*R*(*T*)_*L*_’ is the deterministic part and ‘*R*(*T*)_*U*_*I*_*N*_’ is indeterministic. Under classical extension, the lower indeterminacy value is equal to zero, *i.e. I*_*L*_ = 0, and *I*_*U*_ can be found using (*R*(*T*)_*U*_ − *R*(*T*)_*L*_)/*R*(*T*)_*U*_. For a large number of measurements, the computational algorithm to run on software is as follows:

Step 1: Start program

Step 2: Start an external or main loop from *i* = 20 to *i* <= 100

Step 3: Run internal loop from *I*_*N*_ = *I*_*L*_ = 0 to *I*_*N*_ = *I*_*U*_ (first for *i* = 20)

Step 4: Execute formula for calculating values

*R*(*T*)_*N*_ = *R*(*T*)_*L*_ + *R*(*T*)_*U*_*I*_*N*_; *I*_*N*_ ∈ [*I*_*L*_, *I*_*U*_]

Calculate and draw graph

Step 5: Increment (a specific point as selected by programmer) and go to step 3

Step 6: End internal loop

Step 7: Increment of 5 and go to step 2

Step 8: End external loop

Step 9: End program

The classical and neutrosophic analyses for resistance variance with respect to the change in temperature are shown in Tables 2 and 3, respectively.

**Table 2:** Classical analysis of resistance

Temperature (°C)	Resistance	
	rGO (kΩ)	MWCNTs (kΩ)
20	4.776	0.966
25	4.570	0.965
30	4.339	0.962
35	4.133	0.958
40	3.953	0.955
44	3.722	0.953
50	3.491	0.949
55	3.336	0.947
60	3.234	0.945
65	3.079	0.941
70	2.977	0.937
75	2.925	0.933
80	2.822	0.929
85	2.72	0.925
90	2.54	0.92
95	2.411	0.917
100	2.334	0.912

**Table 3:** Classical analysis of resistance

Temperature (°C)	Resistance	
	rGO (kΩ)	MWCNTs (kΩ)
20	4.499 + 5.063*I*_*N*_; *I*_*N*_ ∈ [0, 0.113]	0.961 + 0.971*I*_*N*_; *I*_*N*_ ∈ [0, 0.010]
25	4.296 + 4.844*I*_*N*_; *I*_*N*_ ∈ [0, 0.113]	0.960 + 0.970*I*_*N*_; *I*_*N*_ ∈ [0, 0.010]
30	4.079 + 4.599*I*_*N*_; *I*_*N*_ ∈ [0, 0.113]	0.957 + 0.967*I*_*N*_; *I*_*N*_ ∈ [0, 0.010]
35	3.885 + 4.381*I*_*N*_; *I*_*N*_ ∈ [0, 0.113]	0.953 + 0.963*I*_*N*_; *I*_*N*_ ∈ [0, 0.010]
40	3.716 + 4.190*I*_*N*_; *I*_*N*_ ∈ [0, 0.113]	0.950 + 0.960*I*_*N*_; *I*_*N*_ ∈ [0, 0.010]
44	3.499 + 3.945*I*_*N*_; *I*_*N*_ ∈ [0, 0.113]	0.948 + 0.958*I*_*N*_; *I*_*N*_ ∈ [0, 0.010]
50	3.282 + 3.700*I*_*N*_; *I*_*N*_ ∈ [0, 0.113]	0.944 + 0.954*I*_*N*_; *I*_*N*_ ∈ [0, 0.010]
55	3.136 + 3.536*I*_*N*_; *I*_*N*_ ∈ [0, 0.113]	0.942 + 0.952*I*_*N*_; *I*_*N*_ ∈ [0, 0.010]
60	3.040 + 3.428*I*_*N*_; *I*_*N*_ ∈ [0, 0.113]	0.940 + 0.950*I*_*N*_; *I*_*N*_ ∈ [0, 0.010]
65	2.894 + 3.264*I*_*N*_; *I*_*N*_ ∈ [0, 0.113]	0.936 + 0.946*I*_*N*_; *I*_*N*_ ∈ [0, 0.010]
70	2.798 + 3.156*I*_*N*_; *I*_*N*_ ∈ [0, 0.113]	0.932 + 0.942*I*_*N*_; *I*_*N*_ ∈ [0, 0.010]
75	2.750 + 3.101*I*_*N*_; *I*_*N*_ ∈ [0, 0.113]	0.924 + 0.938*I*_*N*_; *I*_*N*_ ∈ [0, 0.010]
80	2.653 + 2.991*I*_*N*_; *I*_*N*_ ∈ [0, 0.113]	0.924 + 0.934*I*_*N*_; *I*_*N*_ ∈ [0, 0.010]
85	2.557 + 2.883*I*_*N*_; *I*_*N*_ ∈ [0, 0.113]	0.920 + 0.930*I*_*N*_; *I*_*N*_ ∈ [0, 0.010]
90	2.388 + 2.692*I*_*N*_; *I*_*N*_ ∈ [0, 0.113]	0.915 + 0.925*I*_*N*_; *I*_*N*_ ∈ [0, 0.010]
95	2.266 + 2.556*I*_*N*_; *I*_*N*_ ∈ [0, 0.113]	0.912 + 0.922*I*_*N*_; *I*_*N*_ ∈ [0, 0.010]
100	2.194 + 2.474*I*_*N*_; *I*_*N*_ ∈ [0, 0.113]	0.907 + 0.917*I*_*N*_; *I*_*N*_ ∈ [0, 0.010]

Table 2 presents the classical analysis of the resistance data of both rGO- and MWCNT-based temperature sensors by applying the mean classical formula. It is observed that classical analysis has converted all intervals to fixed points, which are not defining the variance of the interval from minimum to maximum values. We obtained only single values of resistance against specific temperatures. This shows that classical analysis is not much reliable in making decisions and in concluding the solution of the problem. Similarly, Table 3 presents the neutrosophic analysis of the resistance of sensors, from which it is observed that neutrosophic analysis is more reliable as it uses indeterminacy and gives whole information about the resistance variance at specific values of temperature sensors. For example, at 20 °C temperature, the classical value of the resistance of the rGO-based temperature sensor is 4.776 kΩ (a single or fix point value), *i.e. R* (20 °C) = 4.776 kΩ. However, neutrosophic analysis gives the equation *R* (20 °C) = 4.499 + 5.063*I*_*N*_ with the indeterminacy interval *I*_*N*_ ∈ [0, 0.113]. According to the neutrosophic analysis, the value of resistance lies between 4.499 and 5.0631 by putting the indeterminant values.

**Fig. 8:**
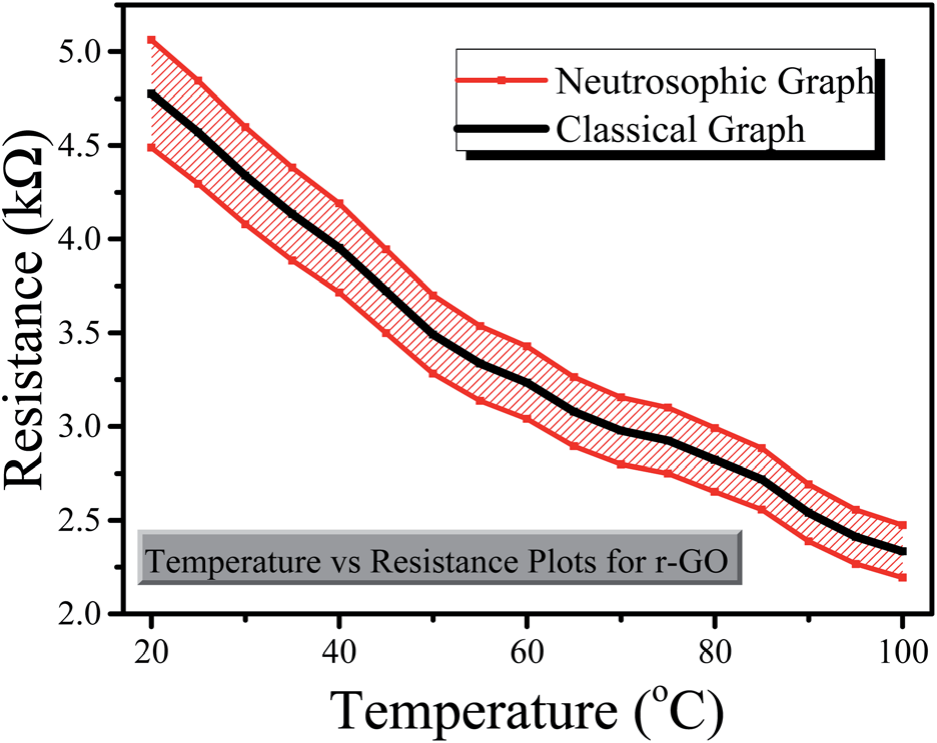
Classical and neutrosophic graphs for the rGO-based sensor.

**Fig. 9:**
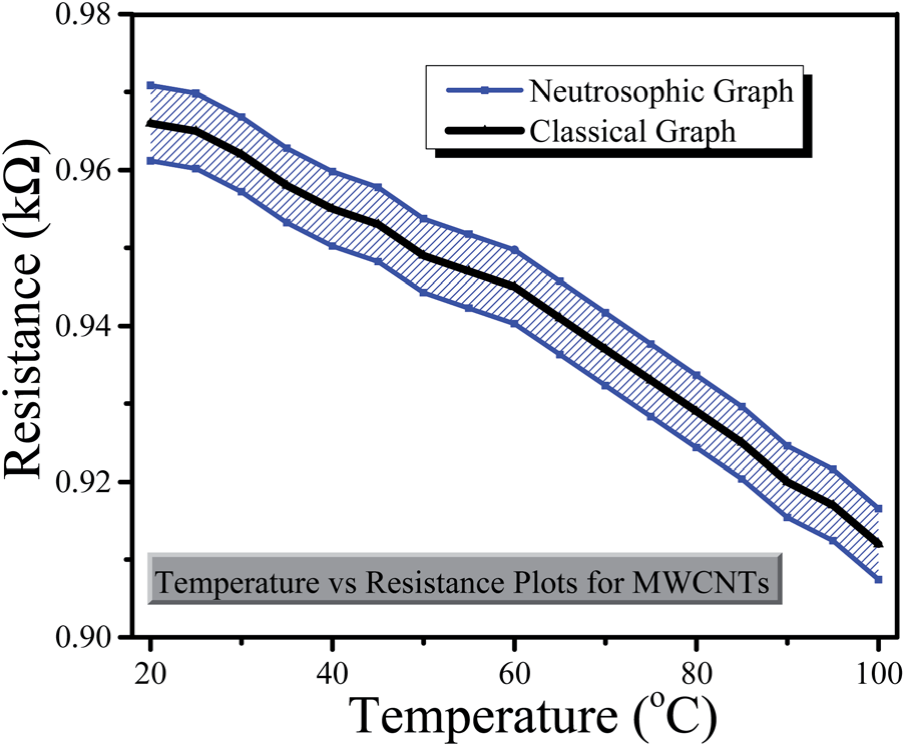
Classical and neutrosophic graphs for the MWCNTs based sensor.

The graphical comparison of both classical and neutrosophic approaches with regard to the resistance of sensors is displayed in Fig. 8 and 9, from which it is clear that the resistance decreased as the temperature increased. Similarly, the graphs also show the comparison between classical and neutrosophic analyses. It was observed that the resistance measured using the rGO-based sensor expressed more data variance. It can easily seen that classical graphs are less flexible and informative to explain the resistances of sensors as these are drawn on fixed point values that are based on single-line graphs. Sometimes, some researchers also use error bars to express variation in the data. Generally, error bar graphs are used to express the error or uncertainty of data.^38^ However, neutrosophic graphs are flexible and informative enough to explain and conclude the problem as these allow us to interact directly with the indeterminant data. Moreover, these graphs show that neutrosophic graphs are more generalized than classical graphs as the neutrosophic graphs also cover the information expressed by classical graphs. The differences between the classical and neutrosophic methods are presented in Table 4.^39^

**Table 4:** Differences between classical and neutrosophic methods

Classical method	Neutrosophic method
Uses the classical formula of mean or average to calculate the value from interval, *i.e.* one can get only a single value for a single value	Uses the neutrosophic equation to calculate the value from interval, *i.e.* one can get numerous values based on the indeterminacy interval
Through this method, interval losses its indeterminacy	This method does not affect the indeterminacy of interval
This uses single-line and error bar graphs	This uses neutrosophic graphs, which cover the whole variation of data
For example, according to the classical method, a statement is only true or only false at a time	For example, according to the neutrosophic method, a statement may be true or false at a time based on its indeterminacy interval

## 4. Conclusion

This work reported the fabrication of temperature sensors based on the carbon materials (rGO and MWCNTs) with high sensitivity (2.869% (kΩ °C^−1^) for rGO and 0.064% (kΩ °C^−1^) for MWCNTs) and their data analyses. The sensors have been fabricated by depositing the carbon material as a sensing layer on a flexible PET substrate between silver electrodes. The carbon materials were characterized by different techniques to study the structure, surface morphology and optical absorption. Also the electrical properties were characterized using an LCR meter at 1 kHz. It was observed that the resistance of both sensors decreased with the increase in temperature because of the semiconductor properties of the carbon materials. Furthermore, the analysis of the electric properties of the sensor was performed using the classical as well as the novel neutrosophic approach. By comparing the methods and formulas, it is concluded that the neutrosophic approach is more effective to analyse the data of temperature sensors, as it is more flexible and informative to take decision.

## Conflicts of interest

There are no conflicts to declare.

## Supplementary Material
